# Dynamic Contrast Microscopic Optical Coherence Tomography As a Novel Method for Assessing Corneal Epithelium During Exposure to Benzalkonium Chloride

**DOI:** 10.1167/tvst.11.5.28

**Published:** 2022-05-27

**Authors:** Gwen Musial, Tabea Kohlfaerber, Martin Ahrens, Hinnerk Schulz-Hildebrandt, Philipp Steven, Gereon Hüttmann

**Affiliations:** 1 University Hospital Cologne, Cologne, Germany; 2 Medical Laser Center Lübeck GmbH, Lübeck, Germany; 3 Institute of Biomedial Optics, University of Lübeck, Lübeck, Germany; 4 Airway Research Center North Member of the German Center for Lung Research, DZL, 22927 Großhansdorf, Germany; 5 Center of Brain, Behavior and Metabolism (CBBM), University of Lübeck, Lübeck, Germany

**Keywords:** toxicity, optical coherence tomography, benzalkonium chloride

## Abstract

**Purpose:**

Microscopic optical coherence tomography (mOCT) has an imaging resolution of 1 µm in all voxel dimensions, but individual epithelial cells are difficult to resolve due to lack of scattering contrast. Adding dynamic contrast processing to mOCT (dmOCT) results in color images that enable visualization of individual cells and possibly give information on cellular function via the calculation of a motility coefficient. We propose this technique as a novel method of evaluating the ocular surface after exposure to a toxic chemical, benzalkonium chloride (BAK).

**Methods:**

Ex vivo cross-section images were acquired with a custom-built, frequency-domain mOCT system. Eyes were explanted from healthy adult C57BL/6 mice and imaged every 30 minutes with five sets of dmOCT scans at each imaging time. Total epithelium and stroma thicknesses were measured from a single mOCT B-scan, and measures of color changes (hue) and the motility coefficient were acquired from dmOCT scans.

**Results:**

After 30-minute exposures to 0.005% BAK, local motility decreased and total epithelium thickness increased compared to controls. For basal epithelium cells, local motility decreased after 60-minute exposures, and the hue shifted red after 90-minute exposures. Stroma thickness did not significantly swell until 150-minute exposures to BAK.

**Conclusions:**

dmOCT allows us to view the behavior of the cornea epithelium under toxic stress due to BAK, revealing parallel swelling of the extracellular matrix and changes in local subcellular motion.

**Translational Relevance:**

The evaluation of the cornea epithelium using dmOCT is helpful to our understanding of the toxic effects of BAK.

## Introduction

In the healthy eye, the transparent cornea refracts light and forms a barrier that defends the eye from chemical, biological, and mechanical insults from the environment. The tight junctions between basal corneal epithelium cells, forming the outermost layer of the cornea, is the primary component of this barrier system.^1^ If the corneal epithelial barrier becomes disrupted from chemicals or disease, the transparency of the cornea can become compromised, leading to diminished vision and increased vulnerability to infection.^2^^,^^3^

Clinically, the cornea epithelium can be assessed by the use of fluorescein stain,^4^ which is applied to the ocular surface and indicates areas of increased permeability in corneal epithelium and cellular damage. This test is simple to perform and is very useful in assessing diseases such as dry eye disease. The cornea epithelium can also be imaged in vivo with contact confocal microscopy to examine the cells of the cornea en face^5^ or with anterior segment optical coherence tomography (OCT)^6^ to cross-sectionally measure the thickness of the cornea.

Experimentally, there are many different human corneal cell culture models, ranging from simple monolayer cultures to three-dimensional models, that have been developed for use in drug toxicity and disease studies. These different models have their advantages and disadvantages and have been thoroughly explored in the review by Rönkkӧ et al.^7^ Ex vivo animal tissue is also used. Corneal opacity, fluorescein retention or permeability, swelling, and other macroscopic changes are markers for tissue damage.^8^^,^^9^ Finally, there are in vivo animal models, and the Draize eye test in rabbits is the gold standard for toxicity studies.^10^^,^^11^

Although there are several methods of evaluating the cornea epithelium in vitro, there is a need for a specific and non-invasive method that can determine the health of corneal epithelium cells in patients. Optical coherence tomography (OCT) is a promising candidate, as devices for corneal imaging are already clinically available; however, these devices lack the resolution and contrast necessary to image the different layers of the corneal epithelium or individual cells. A new technique referred to as microscopic optical coherence tomography (mOCT) leverages the high lateral resolution of microscope objectives and the high axial resolution of ultra-broadband light sources.^12^ When paired with postprocessing algorithms called dynamic contrast intensity,^13^ the resulting dynamic microscopic optical coherence tomography (dmOCT) scans could be the next step forward in corneal toxicity testing and evaluation of diseased corneas. Spectral-domain mOCT has been used to image cornea epithelium ex vivo and has shown results similar to those for confocal microscopy^14^^,^^15^; it has also been used to image the human cornea in vivo.^16^ dmOCT has been demonstrated to create images that closely match the histology of the liver^17^ and esophageal tissue.^18^ Also, a similar technique, full-field OCT with dynamic contrast, has been used to image retinal organoids^19^ and mouse and macaque retinal explants.^20^

The purpose of this study was to image healthy ocular surface tissue with dmOCT and evaluate this imaging method as a way to image ex vivo tissue under a toxic environment. To achieve this, we used a constant exposure to a known toxic chemical, benzalkonium chloride (BAK), and imaged the cornea epithelium over time. Although BAK is a commonly used preservative in eye drops,^21^ it has been shown to cause tear film instability, cytoplasmic damage, apoptosis, mitochondrial dysfunction, and disruption of the corneal epithelium barrier.^22^^–^^24^ The concentration of BAK found in clinically available eye drops ranges from 0.005% to 0.02%.^25^ For these experiments, we chose to expose the whole eye continuously over more than 4 hours to a concentration of 0.005% BAK.

## Methods

All experiments were performed using C57BL/6N mice (Charles River Laboratories, Wilmington, MA). Experiments evaluating the impact of low-concentration BAK over time (21 weeks old), used six mice, and experiments for histology used three additional mice (23 weeks old). Husbandry and all experimental procedures followed approved protocols based on the State Agency for Nature, Environment, and Consumer Protection of the State of North Rhine-Westphalia, Germany and adhered to the ARVO Statement for the Use of Animals in Ophthalmic and Vision Research. After the animals were killed by cervical dislocation, the entire ocular globe was removed, with special care being taken to avoid damaging the cornea epithelium, and glued to a 60-mm tissue culture dish (VMR International, Radnor, PA) with Vetbond Tissue Adhesive (3M Science, St. Paul MN) with the cornea facing up. The tissue culture dishes were filled with warm cell culture media (RPMI 1640 Medium; Thermo Fisher Scientific, Waltham, MA) and placed in an incubator set to 37°C to keep the organs warm between imaging sessions. BAK solution was prepared with 5 mg BAK (Sigma-Aldrich, St. Louis, MO) per 100 mL media, resulting in a 0.005% concentration in the media. All custom software developed to quantify the motility and peak hue values can be made available upon reasonable request.

### Spectral-Domain mOCT Imaging

**Figure 1.**
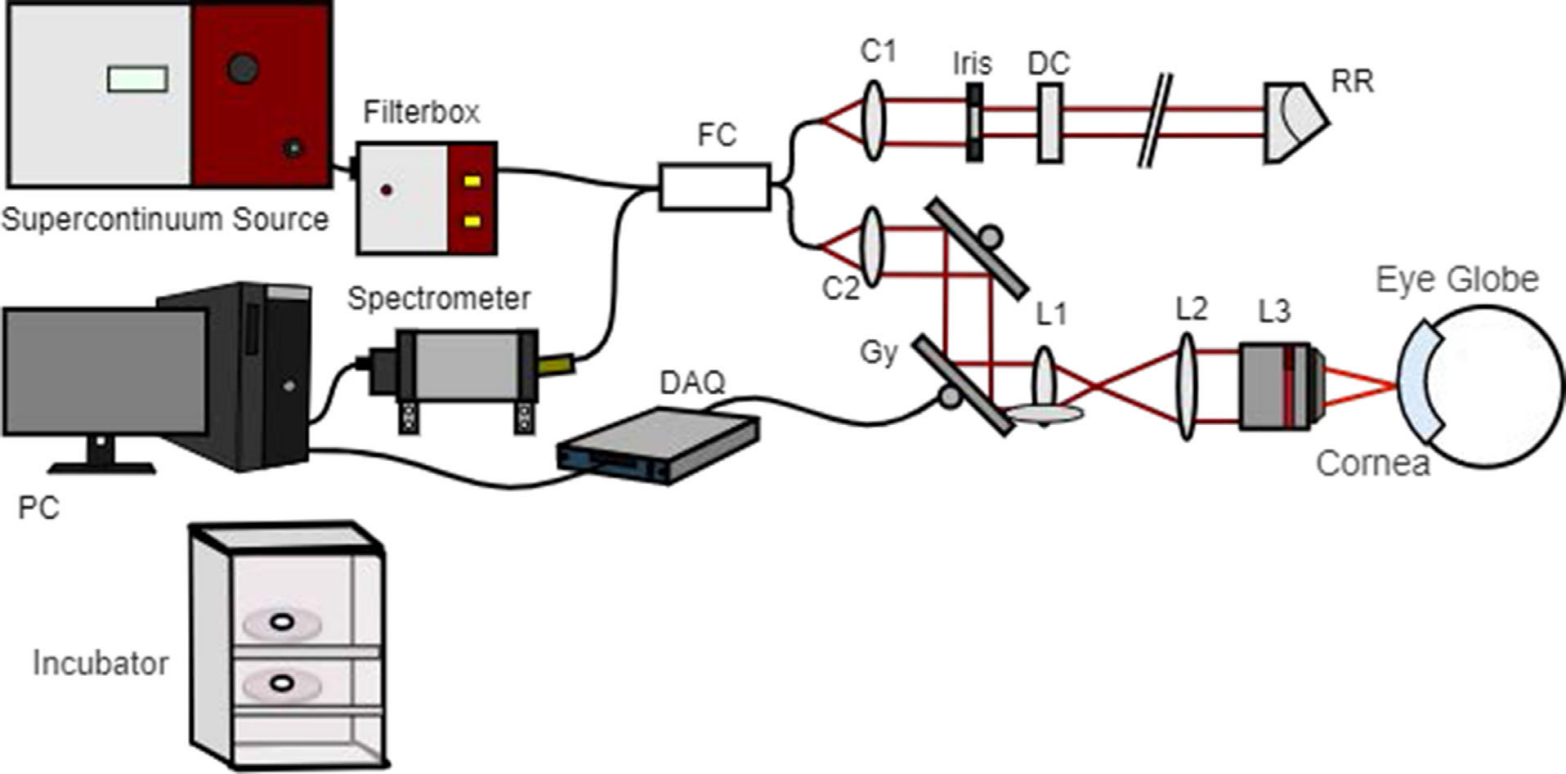
Schematic mOCT setup. Micrometer resolution is obtained by using a supercontinuum light source and a microscope objective (L3). Explanted ocular globes are kept in media under cell culture conditions in an incubator before and after the measurements. Further OCT components include the following: FC, 50/50 fiber coupler; C1/C2, collimators; Gx and Gy, galvanometer mirror scanners; L1 and L2, beam expander lenses; DC, dispersion compensation; RR, retroreflector; DAQ, data acquisition device; and PC, computer for data acquisition and scanning control. Cornea and eye globe are not to scale. Modified from the original version in Münter et al.^17^

Ex vivo cross-section images were acquired with the custom-built Fourier domain mOCT system described in Horstmann et al.^12^ This system utilizes the wavelength range between 550 and 950 nm of a broad-spectrum light source (SuperK; NKT Photonics, Birkerød, Denmark) and an immersion objective (HCX APO L10x/0.30 W U-V-I; Leica, Wetzlar, Germany) to achieve high lateral and axial resolution. Dynamic contrast scans were acquired and processed using the methods described in Münter et al.^17^ Briefly, 150 B-scans with 512 A-scans over a distance of 0.5 mm each were taken for each dynamic contrast scan. With an effective B-scan rate of 111 Hz and a total recording time of 1.35 seconds, the frequency of signal fluctuations was analyzed in the range of up to 55.5 Hz. To create the dmOCT scans, at each voxel the temporal variations in the absolute values of the OCT signal were evaluated. The time series of B-scans was Fourier transformed, and the integral amplitude was calculated in three frequency bands, resulting in an RGB image depicting different time scales of motion activity. Blue represents slow-motion frequencies (0–0.5 Hz); green, medium-motion frequencies (0.5–5 Hz); and red, fast-motion frequencies (5–25 Hz). After the frequencies were split into the three color channels, each channel was normalized from 0 to 1. Then, a moving standard deviation image, with window width of 25 images, was generated, and a histogram of each channel was matched to this image.^26^ Spectral processing of the data was performed using custom programs written in MATLAB R2019b (MathWorks, Natick, MA). Five sets of dynamic contrast scans were acquired at each imaging time point per eye and then averaged for display purposes. All eyes were imaged in normal cell culture media for a baseline point. Then, in the BAK group, the media were replaced with media containing 0.005% BAK as described above. Eyes were removed from the incubator one at a time for dmOCT imaging every 30 minutes over a 5-hour period. The system is illustrated in Figure 1.

### Image Analysis

Analysis was performed on all five dmOCT scans per eye per time point. Two regions of interest were selected for metrics analysis. The total epithelium was manually outlined and the basal epithelium was segmented using custom MATLAB software. The motility coefficient metric was determined to the normalized standard deviation of the intensity of the B-scans over time, averaged over the region of interest (ROI).^27^ This metric was originally described by Oldenburg et al.,^28^ and a detailed illustration of how it was implemented in this manuscript can be found in Supplementary Figure S1. The hue of the dmOCT image gives information on the mean frequency of the motion within an ROI. To determine the peak of the histogram hue within the ROI, the RGB image, created by the frequencies of the dmOCT scan, was first converted to the image format hue, saturation, and value (HSV). Then, the histogram of the hue values was wrapped from hue values from green to green, as hue has a circular scale, and the peaks of the histogram were found. Stroma thickness was measured from mOCT scans manually in ImageJ (National Institutes of Health, Bethesda, MD).^29^ Total epithelium thickness was measured using manual segmentation of the epithelium in dmOCT scans generated using the custom MATLAB software.

### Histology

Histology was done with the corneas of three mice. Two eyes from two different mice were kept as control and were immersed in warm cell culture media (RPMI 1640 Medium; Thermo Fisher Scientific) for 270 minutes and kept in the incubator set to 37°C for the duration of the experiment. The remaining eyes were immersed in the 0.005% BAK and set in the incubator for 30, 60, 90, and 270 minutes. After the allocated time had passed, dmOCT scans were acquired and the 0.005% BAK media disposed of. The dishes were then filled with phosphate-buffered saline for 5 minutes and fixed overnight at 4°C with 5% formaldehyde solution. Next, samples were embedded in paraffin using an embedder (Leica ASP300S; ethanol 80% for 2 × 30 minutes, ethanol 96% for 30 minutes, ethanol 96% for 45 minutes, isopropanol 100% for 2 × 1 hour, isopropanol 100% for 2 × 1.25 hours, Sub-X for 15 minutes, histowax for 3.5 hours, histowax for 2 × 2 hours). The whole globes were cut into 5-µm sections, and the central cornea samples were stained with hematoxylin and eosin (H&E). Then, 40× magnification images were taken with an Olympus (Tokyo, Japan) BX63 microscope equipped with an XM10 monochrome camera.

### Statistical Analysis

Statistical analyses were performed using Prism 8 for Mac (GraphPad Software, San Diego, CA). Significance was analyzed using Student's *t*-test for parametric or Mann–Whitney *U* test for non-parametric datasets. Two-way ANOVA was used to compare the two groups (BAK-treated and control) for each different time points. A value of *P* < 0.05 was considered statistically significant. Shapiro–Wilk tests were performed to test for normality. All data are presented as single or grouped values with mean and standard deviation (SD). All metrics were also measured in the control eye at each time point. These measures were then used to calculate the coefficient of variation and coefficient of repeatability for the thickness, motility coefficient, and hue metrics. The coefficient of variation and coefficient of repeatability of each metric can be found in Supplementary Table S1.

## Results

**Figure 2.**
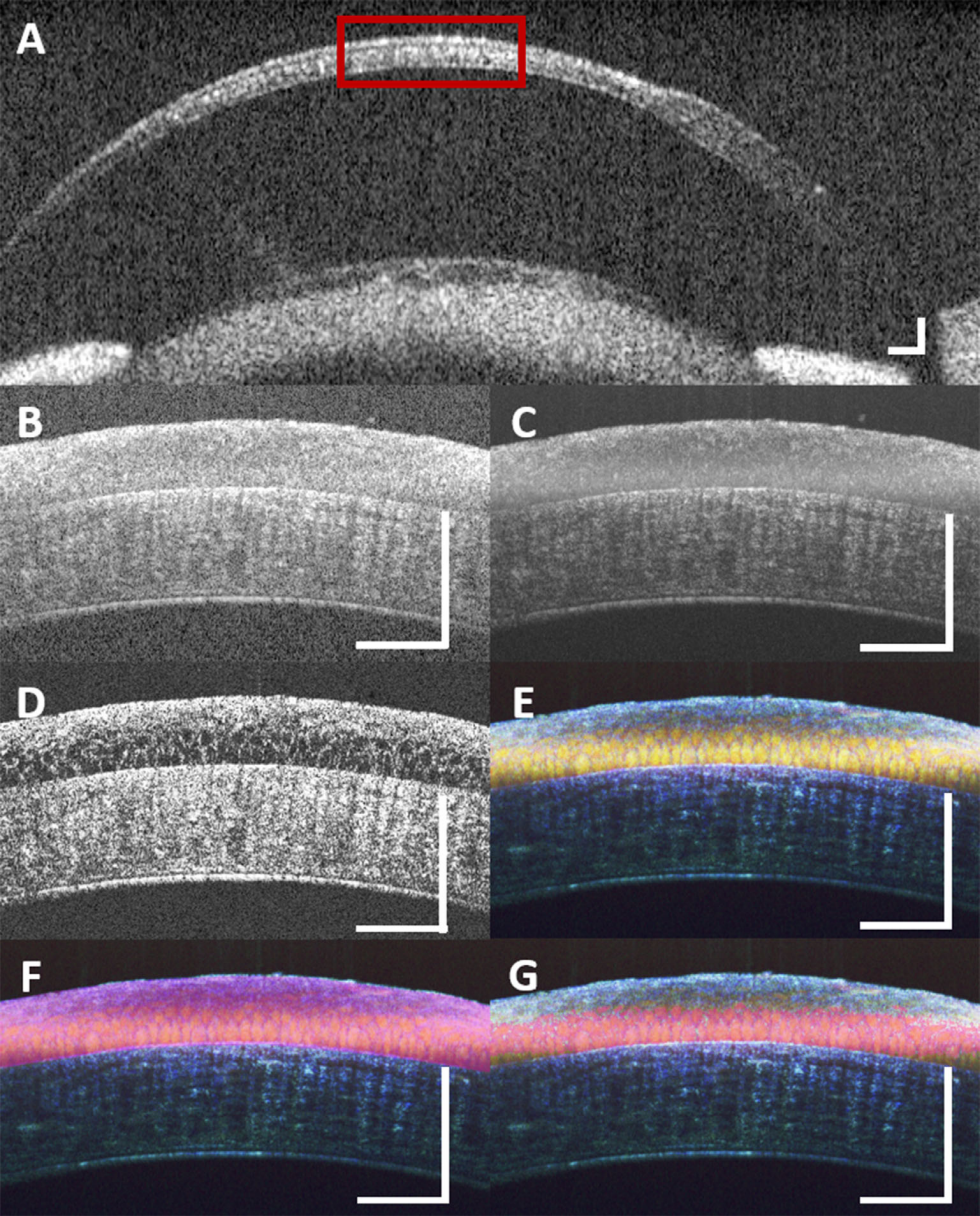
Dynamic contrast mOCT shows individual epithelium cell structure better than conventional OCT and conventional mOCT processing. (**A**) Conventional anterior chamber OCT B-scan in mouse. The *red box* shows the area covered by the mOCT B-scan. (**B**) Single mOCT B-scan. (**C**) Maximum-intensity projection of 150 mOCT B-scans. (**D**) Standard deviation image across 150 mOCT B-scans. (**E**) Dynamic contrast intensity processing over 150 mOCT B-scans (dmOCT) enables visualization of individual epithelial cells and color difference between basal (*yellow*) and superficial (*blue*) layers. (**F**) The *pink overlay* shows the segmentation of the entire corneal epithelium. (**G**) Representative segmentation of the basal epithelium (*pink overlay*). All images (**A**–**G**) were acquired ex vivo in healthy tissue. *Scale bars*: 100 µm.

Individual epithelium cells are visible in the dmOCT scans, and the superficial and basal layers of the epithelium can be delimited. Figure 2A shows a conventional OCT scan of a healthy mouse in vivo with a red box showing the area that is imaged with the mOCT system shown in Figure 2B. In a single B-scan, there is a lot of speckle noise (Figs. 2A, 2B). A stack of 150 B-scans was acquired with the mOCT system as described in the Methods. To create a better structural image of the tissue, it is common to use the conventional processing algorithm of maximum intensity projection to reduce the effect of speckle noise as it fluctuates across the different scans (Fig. 2C). Some of this fluctuation in signal is due to noise, for which the maximum intensity projection accounts; however, some of the fluctuation is created by intracellular motion. To begin to look at functional imaging, algorithms such as standard deviation over the image stack (Fig. 2D) can be used. After this processing, the individual cells seem more apparent compared to a single mOCT B-scan. dmOCT takes this functional imaging further by splitting the fluctuations into different frequency bands (Fig. 2E). The blue channel, or low frequency, depicts the static tissue, and the remaining fluctuation is split into medium-frequency (green) and high-frequency (red) bands. dmOCT results in images that easily discriminate different layers of the cornea and enable visualization of individual cells within the basal layer. Superficial and basal layers of the epithelium are clearly discriminated by different colors. This aids segmentation in two ROIs, the entire cornea epithelium (Fig. 2F) and basal epithelium cells (Fig. 2G), which were used for quantitative evaluation.

**Figure 3.**
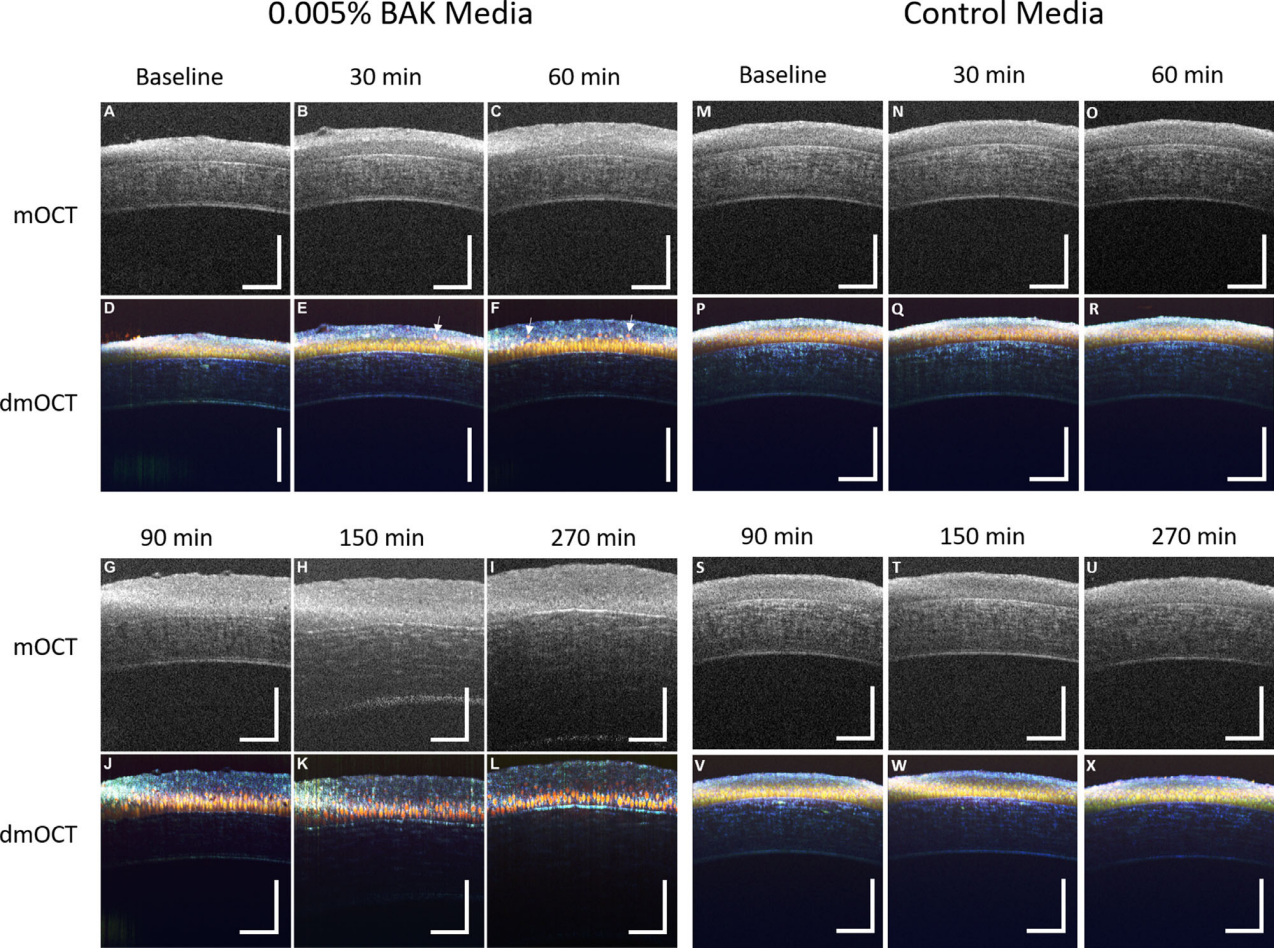
dmOCT in ex vivo cornea during constant exposure to 0.005% BAK. (**A**) Single mOCT B-scan in healthy ex vivo cornea prior to exposure to BAK. *Scan bar*: 100 µm. (**B**, **C**) Single mOCT B-scans after 30-minute and 60-minute exposures to 0.005% BAK show swelling of cornea epithelium. (**D**) dmOCT B-scan in healthy ex vivo cornea prior to exposure to BAK showing superficial and basal epithelium with uniform cell density and tight junctions between basal epithelial cells. (**E**, **F**) dmOCT B-scans after 30-minute and 60-minute exposures to 0.005% BAK showing superficial epithelium cells separating from deeper layer and slight swelling of the cornea epithelium. Examples of these cells separating are highlighted with *white arrows*. (**G**) Single mOCT B-scan after 90 minutes in 0.005% BAK. (**H**) Single mOCT scan showing swelling of cornea stroma after 150-minute exposure to 0.005% BAK. (**I**) Single mOCT B-scan after 270-minute exposure to 0.005% BAK shows severe swelling of stroma. (**J**) dmOCT B-scan after 90 minutes in 0.005% BAK shows a significant red shift in basal epithelial cells. (**K**, **L**) dmOCT B-scans after 120 minutes and 270 minutes in 0.005% BAK show progressive deterioration of cornea epithelium. (**M**–**X**) Corresponding mOCT images of healthy ex vivo cornea at different times after immersion in cell culture media. No changes of scattering intensity and dynamic contrast were visible in the control measurements up to 270 minutes.

Representative images of single mOCT B-scans and dmOCT scans from an eye exposed to 0.005% BAK over time are shown in Figure 3, along with representative images of single mOCT B-scans and dmOCT scans from a control eye which was in tissue culture media over the same amount of time. Both eyes at baseline (Figs. 3A, 3M) have a clear delineation between the epithelium and stroma in the mOCT single B-scan, whereas in the dmOCT images (Figs. 3D, 3P) the individual basal epithelium cells can be seen to be compactly packed and have a yellow hue with the superficial layer on top having a blue hue. After a 30-minute exposure to BAK (Figs. 3B, 3E), the total epithelium swelled, and the superficial layer in the dmOCT appears thicker, with a few cells having a slight red shift. In contrast, after 30 minutes in cell media, the images appear similar to the baseline images. No changes in thickness or hue are visible (Figs. 3N, 3Q). After a 60-minute exposure to BAK (Figs. 3C, 3F), the total cornea epithelium continued to swell, and the cells appear to be detaching from the basal epithelium. Examples of these cells are highlighted in the figure with white arrows. As the time of exposure increased, the cornea epithelium swelled (Figs. 3G, 3J) to a plateau thickness, and the basal cell hue shifted. Then, the stroma swelled (Fig. 3H), and individual cells appear to separate completely as tight junctions seemed to be lost (Figs. 3H, 3K). The stroma continued to swell until the end of the experiment (Fig. 3I), whereas the dmOCT presentation in the cornea epithelium remained very similar between 150 and 270 minutes of exposure to BAK. The appearance of the corneal epithelium and stroma in the control eye remained stable in both mOCT and dmOCT images over the time course of the experiment (Figs. 3M–3X).

**Figure 4.**
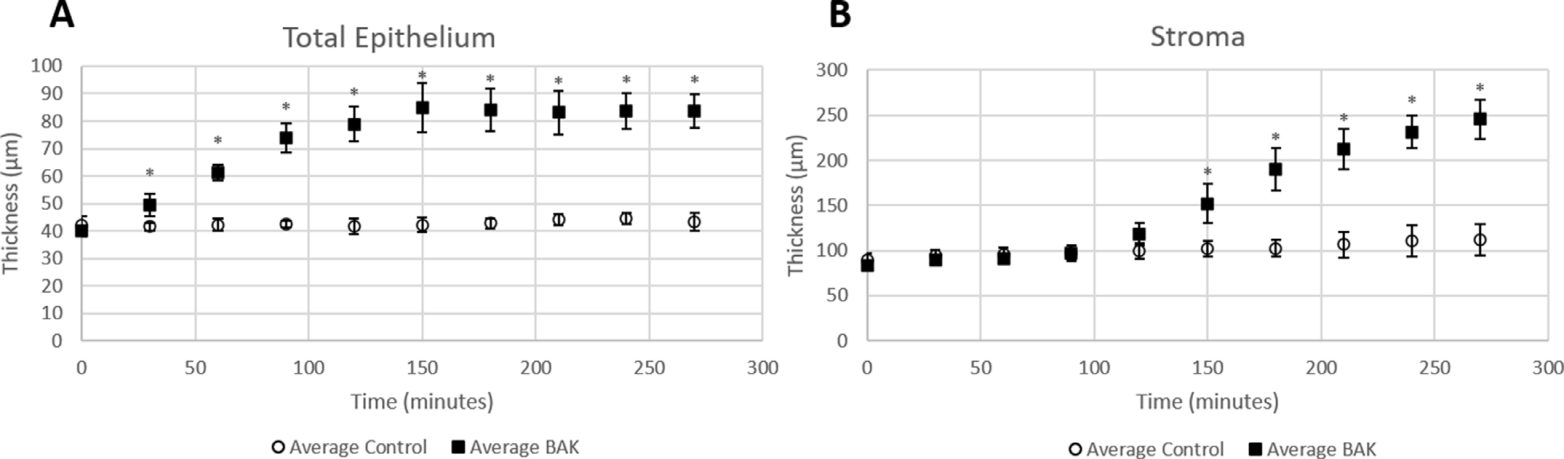
Thickness measurements of the total epithelium and stroma in the central cornea of both control and BAK groups. (**A**) Total epithelium swelled in the 0.005% BAK group after 30-minute exposure and continued to swell until reaching a plateau at 150 minutes. (**B**) Stroma swelling lagged epithelium swelling in the BAK group. Stroma significantly swelled in the BAK group after 150 minutes and continued to swell until the end of the experiment.

Throughout the experiment, the thickness of the epithelium remained constant in the control media (baseline, 42.09 ± 3.18 µm; 270 minutes, 43.36 ± 3.28 µm; *P* > 0.05); the thickness significantly increased in 0.005% BAK after 30 minutes (baseline, 39.91 ± 1.71 µm; 30 minutes, 49.57 ± 4.03 µm; *P* < 0.001) and plateaued after 150 minutes (Fig. 4A). The thickness of the stroma increased slightly over the duration of the experiment in the control media (baseline, 89.93 ± 6.95 µm; 270 minutes, 111.67 ± 17.73 µm; *P* < 0.05). The thickness of the stroma in the eyes in 0.005% BAK media significantly increased compared to control after 150 minutes (baseline, 83.38 ± 4.87 µm; 150 minutes, 151.91 ± 21.43 µm; *P* < 0.05) and continued to increase for the duration of the experiment (Fig. 4B). Both the total epithelium and stroma thickness were more than double in the BAK group by the end of the experiment.

**Figure 5.**
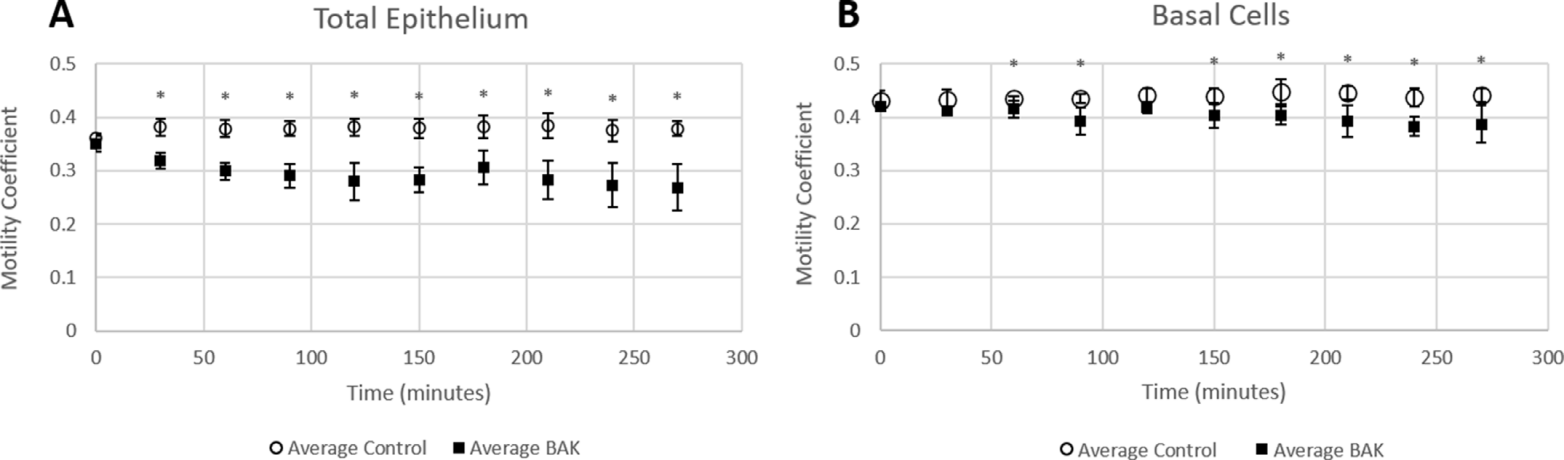
Motility coefficient over time in control eyes and eyes in 0.005% BAK. (**A**) Motility coefficient when measured over the total epithelium first significantly decreased after 30 minutes in 0.005% BAK media. (**B**) Motility coefficient measured only in the basal epithelial cells significantly decreased after 60 minutes in 0.005% BAK.

The motility coefficient was calculated to quantify intracellular changes that caused the color changes in the dmOCT images. It remained constant in the control eyes when measured over the total epithelium (baseline, 0.361 ± 0.007; 270 minutes, 0.379 ± 0.014) and in the basal epithelial cells over the duration of the experiment (baseline, 0.431 ± 0.018; 270 minutes, 0.441 ± 0.013) (Fig. 5). However, the motility coefficient decreased after exposure to 0.005% BAK in both the total epithelium and in the basal epithelial cells. The intracellular motility significantly decreased after 30-minute exposures when measured over the total epithelium (baseline, 0.349 ± 0.015; 30 minutes, 0.319 ± 0.015) (Fig. 5A); however, when measuring only the basal epithelial cells (Fig. 5B), there was no significant decrease until 60-minute exposures to BAK (baseline, 0.420 ± 0.006; 60 minutes, 0.415 ± 0.015).

**Figure 6.**
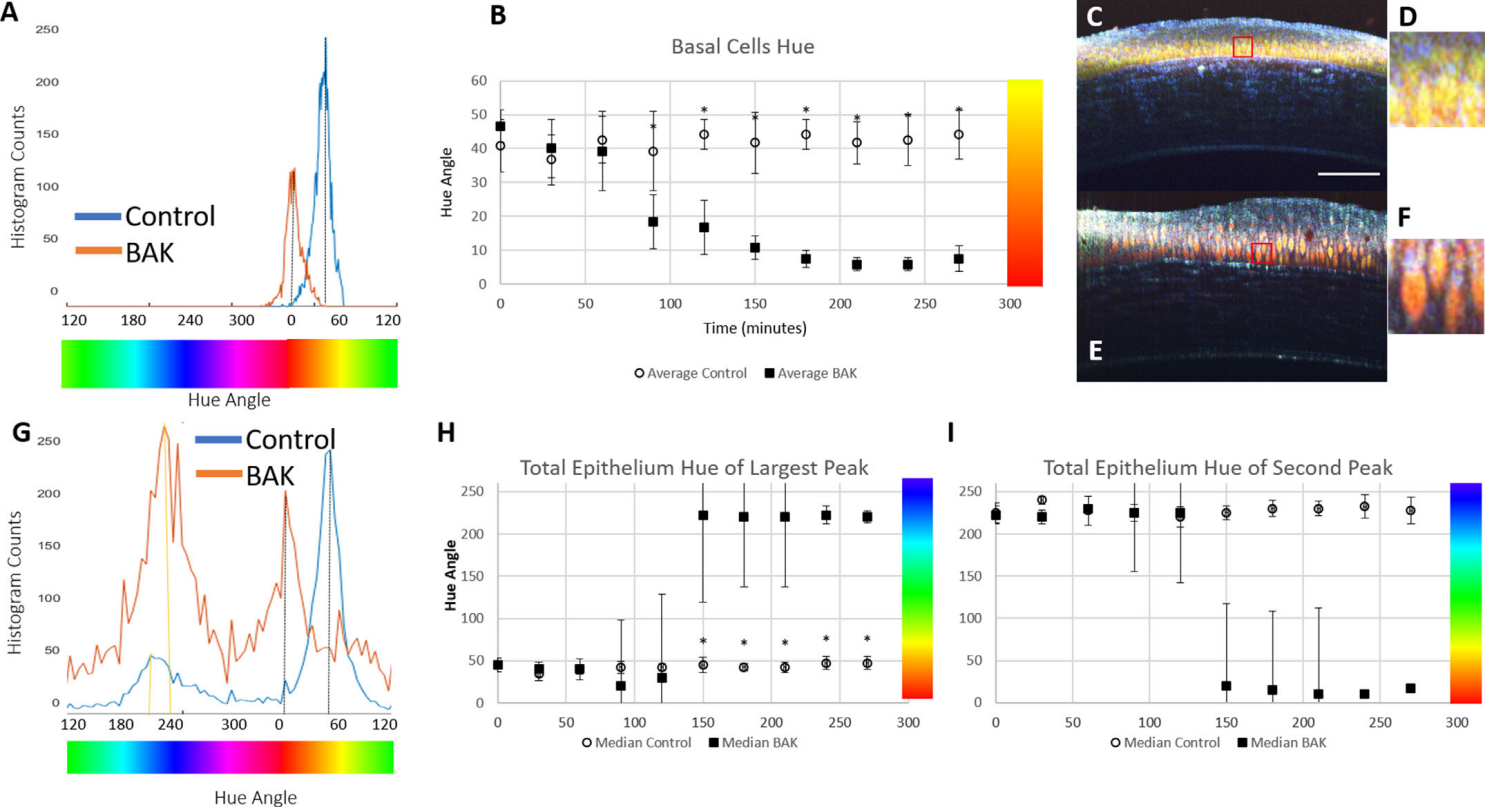
Changes of the hue in the basal and total epithelium of control eyes and eyes exposed to 0.005% BAK. (**A**) Histogram of the hue in the basal epithelial cells in the control eye at 90 minutes (**C**, *blue*) and after 90 minutes exposure to BAK (**E**, *orange*); the peak of the hue histogram for each image is indicated by the *dotted black line*. (**B**) Mean peak hue in the basal epithelial cells over time remained constant in the control eyes but there was a significant shift to red after 90 minutes in the BAK group. (**C**) dmOCT scan in healthy control at 90 minutes, where the *red box* shows excerpt. *Scale bar*: 100 µm. (**D**) The 35-µm section of (**C**) shows the basal epithelium cells in the control conditions. (**E**) dmOCT in 0.005% BAK eye at 90 minutes, where the *red box* shows excerpt. (**F**) The 35-µm section of (**E**) shows the basal epithelium cells after 90-minute exposure to BAK. (**G**) Histograms of the hue in the total epithelium in the control eye at 90 minutes (**C**, *blue*) and after 90-minute exposure to BAK (**E**, *orange*), with the peak of the basal cells hue indicated by the *dotted black line*; the peak of the superficial layer hue for each image is shown with a *dotted yellow line*. (**H**) The largest peak of the hue histogram in the total epithelium over time remained constant in control eyes but there was a significant shift to blue after 180 minutes in the BAK group. (**I**) The second peak in the hue histogram over time remained constant in control eyes corresponding to the superficial layer but there was a shift to red in the BAK group

The color of the dmOCT images is a result of the different frequency of motion of the cells in the image voxels over the duration of the scan. After extended exposure to 0.005% BAK, one visible change is the color shift in the basal epithelium cells over time. Changes of the hue were quantified from the histogram of the hue channel. In Figure 6A, the blue line shows the control eye histogram, and the orange line shows the BAK histogram. The mean peak hue value remained constant in the control eyes over time, whereas there was a notable red shift after 90 minutes in the basal epithelial cells of the BAK group (Fig. 6B). Examples from dmOCT scans in a control eye at 90 minutes (Figs. 6C, 6D) and in an eye after 90-minute exposure to BAK (Figs. 6E, 6F) show the visual differences in the hues of the images. Examination of the histogram of the hue in the total epithelium (Fig. 6G) reveals two peaks in the hue histogram, one corresponding to the basal epithelial cells (yellow hued in healthy control) and an additional peak for the superficial layer (blue hued in healthy control). In the healthy controls (Fig. 6G, blue line) and at the beginning of the experiment in the BAK group, the largest peak in the histogram corresponds to the basal cells and is located in the yellow hue, but as the length of time in the BAK media increased the basal cell hue peak shifted to red and decreased (Fig. 6G, orange line, Fig. 6H). The second peak in the histogram that corresponds to the superficial layer (blue hue) increased as the cornea swelled and the superficial layer thickened (Fig. 6I).

**Figure 7.**
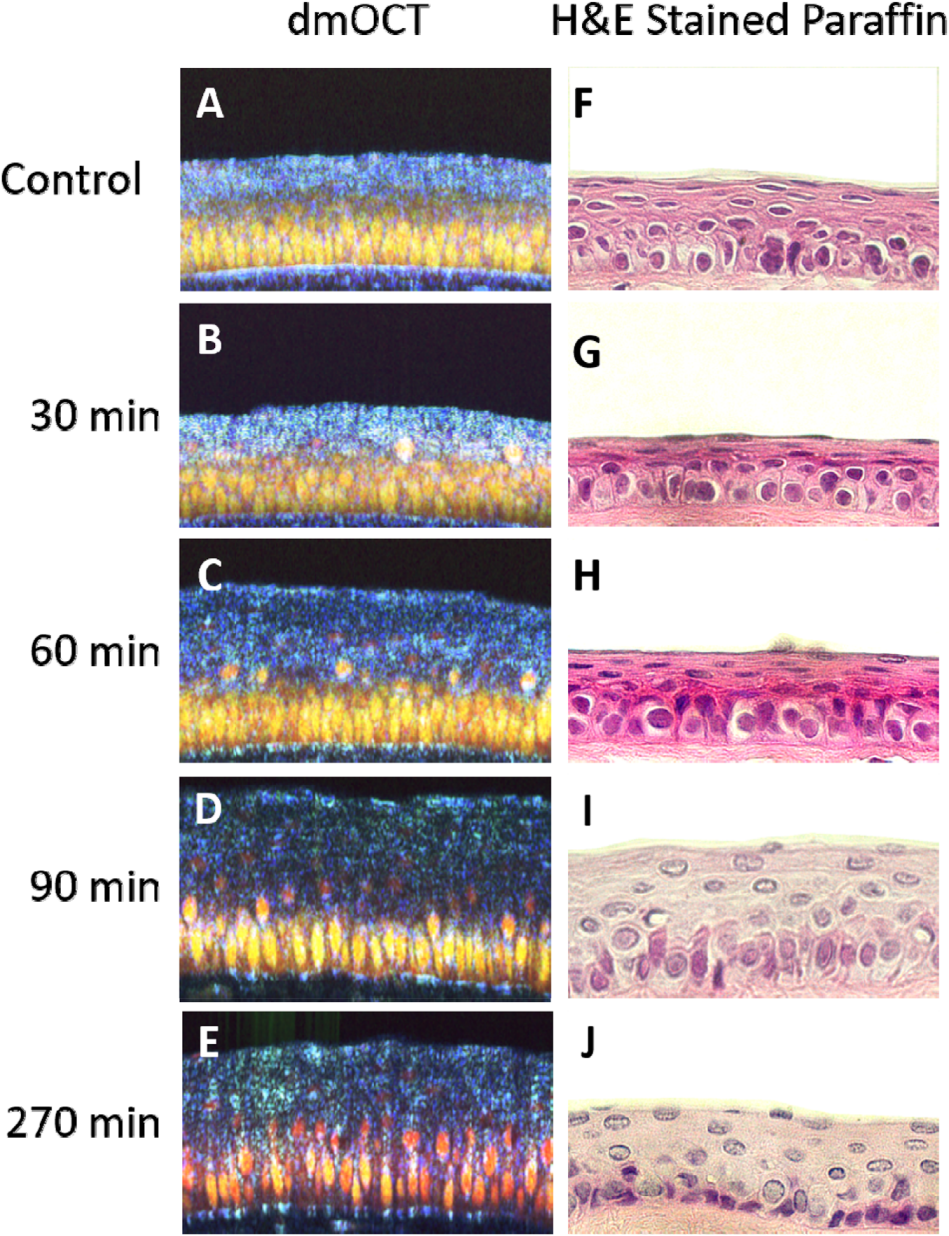
Comparison of dmOCT images to H&E-stained images after prolonged exposure to 0.005% BAK. (**A**) dmOCT B-scan in healthy ex vivo cornea 270 minutes after immersion in cell culture media. (**B**–**E**) dmOCT B-scans in healthy ex vivo cornea 30, 60, 90, and 270 minutes after immersion in 0.005% BAK cell culture media. (**F**) H&E-stained sample of healthy ex vivo cornea 270 minutes after immersion in cell culture media. (**G**–**J**) H&E-stained sample of healthy ex vivo cornea 30, 60, 90, and 270 minutes after immersion in 0.005% BAK cell culture media. All images are at the same scale and have the dimensions of 250 µm × 150 µm in width and height.

The effects of prolonged exposure to 0.005% BAK can also be seen in histological slides stained with H&E (Fig. 7). The H&E-stained corneas show the same distinct bilayer structure that was visible in dmOCT as yellow-hued basal cells and a blue-hued superficial layer. In the H&E-stained cornea, the basal cells are round and evenly packed but the superficial cells are oval or flat shaped and have slightly elongated nuclei (Figs. 7A, 7F). After 30 and 60 minutes of 0.005% BACK exposure, it became difficult to discern changes in the H&E-stained tissue and the dmOCT scans (Figs. 7B, 7C, 7G, 7H), although the motility coefficient significantly decreased. After 90 minutes of 0.005% BAK exposure, both the dmOCT and H&E-stained images show changes in the superficial and basal layers. The cells appear to be lifting in the superficial layer of both images, and both have slightly elongated cell shapes in the basal layer (Figs. 7D, 7I). At the conclusion of the experiment, after 270 minutes in 0.005% BAK media, there were significant changes in the dmOCT image and metrics, whereas the H&E stained image appears similar to the image from the sample which was only exposed to 90 minutes of 0.005% BAK (Figs. 7E, 7J). Tissue thickness in the two imaging modalities cannot be directly compared due to the shrinkage that occurs in the fixation process during preparation of the H&E-stained tissue.

## Discussion

This study utilized dmOCT to image ex vivo healthy mouse cornea in normal culture media and after constant exposure to a low concentration of BAK (0.005%). To the best of our knowledge, this is the first study to characterize corneal damage after BAK exposure by OCT. The basis of the dynamic contrast signal in dmOCT has been evaluated by several research groups and is likely to originate from motion from mitochondrial action, or small vesicles such as vesicles or lysosomes, making it an interesting potential tool to evaluate cellular toxicity.^18^^,^^30^^–^^33^ The resulting color images from dmOCT scans are richly contrasted. Based on this tissue-specific color contrast, we propose the new metric of peak hue position as a method to evaluate the behavior of tissue imaged with dmOCT. Other metrics evaluated in this study of motility and thickness have been used by other research groups as a measure of cellular health and as a measure of corneal damage.^34^^–^^37^ The methods used in this study corroborate other studies that show that BAK has a toxic effect on the cornea epithelium and stroma, even at low concentrations, when there is a long exposure time.^11^^,^^21^^–^^23^^,^^38^^–^^40^

Microscopic OCT has been adapted to image the human cornea non-invasively in vivo,^41^ so the methods presented in this study are early steps toward non-invasively demonstrating the toxic effects of substances on the corneal epithelium. However, in vivo samples will be influenced by respiration or heart pulsation, which interfere with the dynamic contrast. For this reason, further work is needed before direct application of this technique.

One response of the cornea to BAK was swelling, probably caused by the loss of epithelial integrity. Damage to the endothelium could cause swelling of the stroma, but assessment of the endothelium was not the aim of this study, and we could not image the endothelium with the current scanning parameters. Additionally, these experiments were performed on entire excised globes and not isolated corneas; therefore, it is more likely that the stroma encountered the BAK toxic effects prior to the endothelium. The thickness measurements of the healthy cornea epithelium and stroma obtained using the mOCT B-scans closely match values from the literature. In our control eyes and baseline BAK group eyes, thickness measures of the central cornea epithelium (42.09 ± 3.17 µm and 39.90 ± 1.71 µm, respectively) and central cornea stroma (89.93 ± 6.95 µm and 83.38 ± 4.87 µm, respectively) are within the range reported by Henriksson et al.^42^ for healthy adult C57BL/6 mice (40.59 ± 5.8 µm and 90.88 ± 14.8 µm, respectively). Other in vivo studies that have measured the effect of BAK application on cornea thickness in animal models have used confocal microscopy to measure total cornea thickness. These studies did not evaluate if there were epithelium thickness changes prior to stroma thickness changes as our results show but did find that overall application of high concentration (0.1%–0.25%) BAK resulted in thickening of the central cornea.^38^^,^^43^ Interestingly, a human in vivo study utilizing OCT measurements used multiple regression analysis to determine that glaucoma patients using eye drops containing BAK was significantly correlated with having thinner central cornea epithelium thickness.^37^ However, their monotherapy subgroup data suggested that the use of BAK-containing eyedrops did not induce alterations in epithelial thickness.^37^ The normal application of low-concentration BAK may not induce thickness changes, so functional or more specific assays are needed to see if there are long-term toxic effects. In particular, the hue shift had a much larger difference between the control and BAK groups compared to the motility coefficient decrease.

The measures of decreased motility and hue shift match with what is known about the general effect of toxic substances on cellular metabolism and the effects on the structure of the cornea.^44^ In a general toxic insult, a loss of membrane integrity, which could be occurring during swelling of the cornea epithelium, leads to rapidly decreasing levels of ATP in the cells. Additionally, toxicity can result from different cellular events, including changes in enzymatic activity and morphology, as well as cellular detachment.^45^^,^^46^ Therefore, the decrease in cellular activity during exposure to BAK is to be expected, as BAK has a known inhibitory effect on adenosine triphosphate synthesis.^22^ In in vivo animal studies, the basal epithelial cells were found to be disorganized and apoptotic after long-term application of BAK.^47^^,^^48^ In vitro cellular studies have found cell detachment of cultured corneal epithelial cells at a BAK concentration of 0.005% after 30 minutes of exposure.^40^ In our ex vivo study, there was significant swelling of the cornea epithelium after 30-minute exposures to 0.005% BAK, and, in the dmOCT scans where individual cells can be visualized, the top basal epithelial cells appear to begin to lift and separate.

Although the cross-sectional view of the cornea provides valuable information on the interaction between the cornea epithelium layers and the epithelium/stroma border, it does not enable full visualization of cell morphology. An en face view from a volume mOCT would enable visualization of the three-dimensional cellular structure. Currently, the mOCT system configuration is not fast enough to produce these scans with the number of B-scans required for the dynamic contrast, but this technical limitation has been overcome by other groups.^49^ Another application of this technology could be in scanning three-dimensional multilayered in vitro cornea models.^50^ One of the key benefits of the ex vivo model used in this study is that the model shows the whole cornea, including the multilayered epithelium and stroma, and better matches the in vivo condition. However, constant immersion of the cornea in media with a toxic substance does not mimic the physiological conditions of an in vivo model and is more closely related to an in vitro model. A more realistic experimental setting would be to use dmOCT to scan animals longitudinally after application of BAK as eyedrops. The cells are protected with the action of the eyelids, the pre-ocular mucin layer of the tear film, and the permanent renewal of ocular surface epithelia as these tissues have high regeneration.^21^ One challenge for translating the dmOCT scan method to in vivo measurements is tissue motion. For this study in ex vivo tissue, we could be sure that there was no bulk motion due to tissue movement, but in an in vivo study motion due to breathing or the heart beating would have to be controlled for or filtered out. In the postprocessing of the dmOCT scans, the individual B-scans would require alignment based on the phase information in the OCT scans. Further work should be done to determine if this can compensate for large motion artifacts.

## Supplementary Material

Supplement 1

Supplement 2
