# Supplementary figures and images for: Dynamic Contrast Microscopic Optical Coherence Tomography As a Novel Method for Assessing Corneal Epithelium During Exposure to Benzalkonium Chloride
Source: Transl Vis Sci Technol. 2022 May 27;11(5):28. doi: 10.1167/tvst.11.5.28 (PMC9145126; doi:10.1167/tvst.11.5.28)

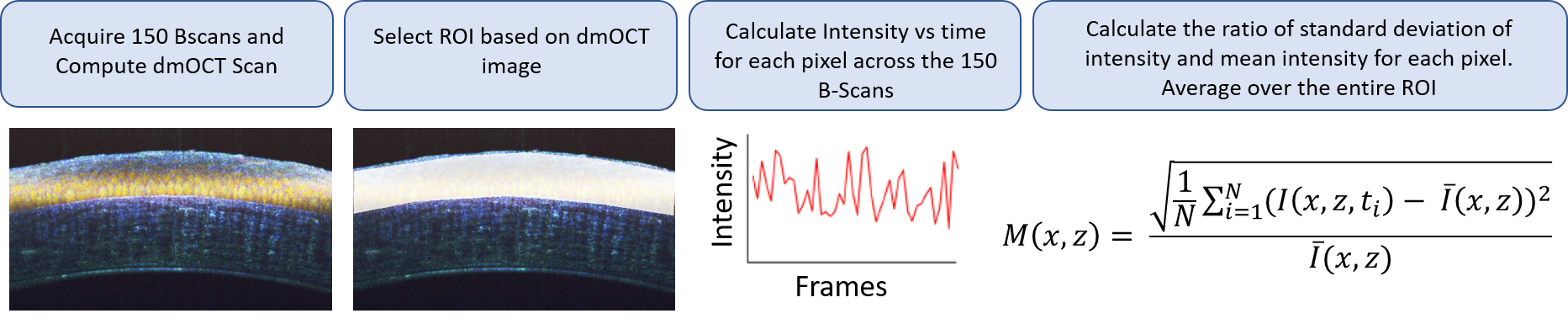

Supplement: Supplement 1 [file tvst-11-5-28_s001.tif]
