# Supplementary material for: Dynamic Contrast Microscopic Optical Coherence Tomography As a Novel Method for Assessing Corneal Epithelium During Exposure to Benzalkonium Chloride
Source: Transl Vis Sci Technol. 2022 May 27;11(5):28. doi: 10.1167/tvst.11.5.28 (PMC9145126; doi:10.1167/tvst.11.5.28)
Supplement: Supplement 2 [file tvst-11-5-28_s002.pdf]

The coefficient of variation was computed by the ratio of the standard deviation of the average of all eyes measurement over for each time point in the control group, over the average of all the measures over time, and multiplying by 100. The coefficient of repeatability was calculated by first computing the within subject standard deviation over time, multiplying this by 2.77, then taking the average of all six control eyes.

Table S1. Measures of reproducibility in control eye data

	Coefficient of Variation (CV)	Coefficient of Repeatability (CR)
Formula	$\frac{\sigma}{\bar{x}} * 100$	within-subject standard deviation (S_W)*2.77
Epithelium Thickness	4.8 ± 1.4	5.7µm ± 1.7µm
Stroma Thickness	7.1 ± 3.6	20.4µm ± 12.2µm
Basal Motility	2.098 ± 0.374	0.025 ± 0.005
Total Motility	3.109 ± 0.593	0.033 ± 0.006
Peak Hue Basal	13 ± 5	15 ± 5
Primary Hue Total	11 ± 5	12 ± 5
Secondary Hue Total	5 ± 2	32 ± 13
